# Prevalence of human papillomavirus infection of 65,613 women in East China

**DOI:** 10.1186/s12889-019-6487-9

**Published:** 2019-02-11

**Authors:** Yanmei Ge, Shanliang Zhong, Meng Ren, Yanping Ge, Yuan Mao, Peng Cao

**Affiliations:** 1 Laboratory of gene amplification, Kingmed Diagnostics, Nanjing, 210043 China; 2 Center of Clinical Laboratory Science, Jiangsu Cancer Hospital & Jiangsu Institute of Cancer Research & The Affiliated Cancer Hospital of Nanjing Medical University, Baiziting 42, Nanjing, 210009 China; 3 Institute of Tropical Medicine Third Military Medical University, Chongqing, 400037 China; 4 School of Bioligical Science and Food Engineering, Chuzhou University, Chu zhou, 239000 China

**Keywords:** Human papillomavirus, Prevalence, Cervical cancer, Vaccines

## Abstract

**Background:**

The infection with human papillomavirus (HPV) is the major cause of genital disease. This study assessed the prevalence and genotype of HPV among outpatient women and healthy women in Jiangsu, East China.

**Methods:**

A total of 65,613 women aged 16–85 years were recruited from Nanjing Kingmed Diagnostics, including 45,736 outpatients and 19,877 healthy women. The cervico-vaginal cells were collected and then HPV types were detected using the Tellgenplex™ HPV DNA Test.

**Results:**

The overall HPV prevalence was 17.7% for outpatients and 10.6% for healthy women. 13.7% outpatients were infected with a single HPV type and 4.0% were infected with multiple HPV types. Regarding healthy women, 8.5 and 2.1% were infected with single and multiple HPV types, respectively. The two most commonly detected HPV types were HPV 16 and 58 regardless of single- or multiple-type infection or source of the participants. HPV16 + 58 was the most commonly identified multiple genotype in outpatients, while HPV16 + 52 was frequently detected in healthy women. Highest prevalence rate was found in outpatients aged < 20 years and ≥ 60 years.

**Conclusions:**

This study revealed the prevalence characteristics of HPV in both outpatient women and healthy women in Jiangsu province.

**Electronic supplementary material:**

The online version of this article (10.1186/s12889-019-6487-9) contains supplementary material, which is available to authorized users.

## Background

The infection with human papillomavirus (HPV) is strongly associated with genital disease. Type-specific persistent infection with high-risk (HR) HPV is the major reason of cervical squamous intraepithelial lesions as well as cervical cancer [1–3]. According to its relationship with cervical cancer and precancerous lesions, HPV genotypes are divided into HR-HPV and low-risk (LR) HPV genotypes [4]. These different HPV genotypes have different pathogenicity [5]. The HR-HPV genotypes may lead to cervical carcinogenesis, while the LR-HPV are associated with benign or low-grade changes in cervical cells, such as genital warts [6, 7].

There are many reports on the prevalence of HPV in different populations. The prevalence of HPV infection varies greatly by country, region within country, and population [8, 9]. Therefore, understanding the prevalence characteristics of HPV in different regions is very important for the early detection and prevention of local cervical cancer. In the present study, we made a comprehensive analysis of the prevalence characteristics of HPV in both outpatient women and healthy women who were recruited from Jiangsu, China.

## Methods

### Study population

The women who received HPV screening at Nanjing KingMed Diagnostics between January of 2016 and May of 2017 were recruited. We excluded the women who did not have valid testing results (e.g., the unsuccessful test as a result of unqualified sampling) or who were not the residents of Jiangsu Province. Finally, we enrolled 65,613 women aged 16–85 years, including 45,736 outpatients and 19,877 healthy women who received routine examination. All the data were retrospectively collected from Laboratory Information Management System of Nanjing KingMed Diagnostics.

### Specimen collection

All the participants were asked to do not douche or use tampons or vaginal creams, deodorants, or medications for 2 days before the test. The participants were not in menstruation period and refrained from sex for 48 h before the test. The cervico-vaginal cells were collected from the transformation zone of the uterine cervix by a gynecologist or a trained physician using a standard cytobrush, then suspended in a standard transport medium (STM) for the Tellgenplex™ HPV DNA Test (Tellgen Life Science Co., Shanghai, China) and stored at 4 °C until analyzed (24 h–72 h) [10].

### HPV genotyping

HPV types were detected using the Tellgenplex™ HPV DNA Test (Tellgen Life Science Co. Ltd., Shanghai, China). It could identify the 27 most common HPV genotypes including 14 HR-HPV genotypes (HPV 16, 18, 31, 33, 35, 39, 45, 51, 52, 56, 58, 59, 66, 68) and 13 LR-HPV/undetermined risk (UR-) HPV genotypes (6, 11, 26, 40, 42, 43, 44, 53, 61, 81, 82, 83). Briefly, DNA was extracted, then 100 ng HPV DNA was used as template and added into 20 μl reaction mixture that includes 10 μl PCR premixed solution, 0.8 μl Taq DNA Polymerase and 5 μl primer mixture.

### Statistical analysis

The SPSS version 19.0 (IBM, Armork, NY, USA) was used to perform all statistical analyses. HPV prevalence rate was estimated by a proportion and summarized as a percentage, with an exact binomial 95% confidence interval (95% CI). Prevalence estimates are presented overall and by age (< 20 years, 20–29 years, 30–39 years, 40–49 years, 50–59 years and ≥ 60 years). The chi-squared (*x*^*2*^) was used to test for departure from homogeneity in prevalence across all the 6 age groups. The prevalence was estimated separately for outpatients and healthy women. We used data of healthy women to estimate percentage of women who will be protected by the vaccines targeting different HPV types. *p* < 0.05 was considered statistically significant.

## Results

### The overall prevalence of HPV infection

In the 45,736 outpatient women, 8081 participants (17.7, 95% CI: 17.3–18.0%) were infected with at least one HPV type, including 6253 participants (13.7, 95% CI: 13.4–14.0%) infected with a single HPV type and 1828 participants (4.0, 95% CI: 3.8–4.2%) infected with multiple HPV types (*P* < 0.001). The HR-HPV prevalence rate (15.7, 95% CI: 15.3–16.0%) was higher than the LR/UR-HPV rate (7.4, 95% CI: 7.2–7.7%) (Table 1).

**Table 1:** The prevalence of HPV infection in all the specimens

					Multiple infection (%)	
Participants	Total (%)	HR-HPV (%)	LR/UR-HPV (%)	Single (%)	Dual	3 or More
Outpatient women	8081(17.7)	7160(15.7)	3405(7.4)	6253(13.7)	1336(2.9)	492(1.1)
Healthy women	2110(10.6)	2002(10.1)	611(3.1)	1696(8.5)	335(1.7)	79(0.4)
*P*	0	0	0	0	0	0

Regarding 19,877 healthy women, 8.5% (95% CI: 8.1–8.9%) were infected with single HPV subtype, whereas 2.1% (95% CI: 1.9–2.3%) were infected with multiple HPV subtypes (*P* < 0.001). HR-HPV and LR/UR-HPV infection was detected in 2002 (10.1, 95% CI 9.7–10.5%) and 611 (3.1, 95% CI: 2.8–3.3%) healthy women, respectively (Table 1). We estimated percentage of women who will be protected by the vaccines targeting different HR-HPV types. The estimates are presented in Additional file 1: Table S1. We further analyzed the distribution of HPV types in HPV-positive specimens. The top ten HPV types are presented in Table 2. The two most commonly detected HPV types were HPV 16 and 58 regardless of single- or multiple-type infection or source of the participants.

**Table 2:** The prevalence of the top ten frequences of HPV type of the single/multiple infection in the outpatient and healthy women

Infection Type	Outpatient women			Healthy women		
	HPV Type	n	Prevalence rate (95% CI)	HPV Type	n	Prevalence rate (95% CI)
Single infection	HPV16	1018	12.6 (11.9–13.3)	HPV58	194	9.2(8.0–10.4)
	HPV58	702	8.7(8.1–9.3)	HPV16	187	8.9(7.6–10.1)
	HPV52	588	7.3(6.7–7.8)	HPV52	146	6.9(5.8–8.0)
	HPV53	338	4.2(3.7–4.6)	HPV53	122	5.8(4.8–6.8)
	HPV39	321	4.0(3.5–4.4)	HPV39	105	5.0(4.0–5.9)
	HPV18	285	3.5(3.1–3.9)	HPV61	105	5.0(4.0–5.9)
	HPV61	278	3.4(3.0–3.8)	HPV56	86	4.1(3.2–4.9)
	HPV56	256	3.2(2.8–3.5)	HPV81	82	3.9(3.1–4.7)
	HPV81	247	3.1(2.7–3.4)	HPV59	74	3.5(2.7–4.3)
	HPV51	227	2.8(2.4–3.2)	HPV51	73	3.5(2.7–4.2)
Multiple infection	HPV16	415	5.1(4.7–5.6)	HPV16	98	4.6(3.7–5.5)
	HPV58	406	5.0(4.5–5.5)	HPV58	94	4.5(3.6–5.3)
	HPV52	363	4.5(4.0–4.9)	HPV53	75	3.6(2.8–4.3)
	HPV53	266	3.3(2.9–3.7)	HPV52	65	3.1(2.3–3.8)
	HPV39	243	3.0(2.6–3.4)	HPV39	60	2.8(2.1–3.6)
	HPV56	207	2.6(2.2–2.9)	HPV61	56	2.7(2.0–3.3)
	HPV61	205	2.5(2.2–2.9)	HPV56	48	2.3(1.6–2.9)
	HPV18	191	2.4(2.0–2.7)	HPV66	48	2.3(1.6–2.9)
	HPV81	191	2.4(2.0–2.7)	HPV51	43	2.0(1.4–2.6)
	HPV51	188	2.3(2.0–2.7)	HPV43	42	2.0(1.4–2.6)

### The prevalence of multiple HPV infection

In the outpatient women, there were 1336 participants infected with dual HPV types and 492 participants infected with three or more HPV types (Table 1). In healthy women, there were 335 participants infected with dual HPV types and 79 participants infected with three or more HPV types (Table 1). Table 3 presents the top ten combinations of HPV types in the participants. The top three most common combinations of the types were HPV16 + 58, HPV52 + 58 and HPV39 + 52 for outpatient women and HPV16 + 52, HPV52 + 58 and HPV39 + 58 for the healthy women (Table 3). The detailed distributions of multiple HPV infection are presented in Additional file 2: Table S2.

**Table 3:** The top ten multiple HPV infection mode in participants

Outpatient women			Healthy women		
Combination of types	n	Prevalence rate (95% CI)	Combination of types	n	Prevalence rate (95% CI)
16 + 58	44	2.4(1.7–3.1)	16 + 52	11	2.7(1.1–4.2)
52 + 58	33	1.8(1.2–2.4)	52 + 58	11	2.7(1.1–4.2)
39 + 52	21	1.1(0.7–1.6)	39 + 58	9	2.2(0.8–3.6)
52 + 61	19	1.0(0.6–1.5)	53 + 58	8	1.9(0.6–3.3)
16 + 39	19	1.0(0.6–1.5)	16 + 58	8	1.9(0.6–3.3)
18 + 58	18	1.0(0.5–1.4)	35 + 53	6	1.4(0.3–2.6)
16 + 56	16	0.9(0.4–1.3)	16 + 51	6	1.4(0.3–2.6)
16 + 33	15	0.8(0.4–1.2)	16 + 35	5	1.2(0.2–2.3)
16 + 53	15	0.8(0.4–1.2)	16 + 66	5	1.2(0.2–2.3)
33 + 58	15	0.8(0.4–1.2)	58 + 61	5	1.2(0.2–2.3)

### The prevalence of HPV infection in different age groups

We analyzed the distribution of positive HPV results in different age groups. Our results showed that the under 20 age group owned the highest rate of HPV infection (36.7, 95% CI: 32.2–41.2%), followed by the over 60 age group (25.6, 95% CI: 23.3–27.9%), while the 30–39 age group had the lowest prevalence rate (17.0, 95% CI: 16.4–17.6%) in the outpatient women (*P* < 0.001). In the healthy women, people aged 50–59 years had the highest prevalence rate (12.4, 95% CI: 11.1–13.6%) and the 30–39 age group had the lowest prevalence rate (9.9, 95% CI: 9.3–10.6%) (*P* < 0.001) (Table 4).

**Table 4:** Prevalence of HPV infection in different age groups

	Outpatient Women		Healthy Women	
Age groups	n (postive/total)	Prevalence rate (95% CI)	n (postive/total)	Prevalence rate (95% CI)
< 20	164/447	36.7(32.2–41.2)	0/0	0
20–29	1733/9886	17.5(6.8–18.3)	327/3098	10.6 (9.5–11.6)
30–39	2325/13671	17.0(16.4–17.6)	822/8293	9.9 (9.3–10.6)
40–49	2468/12339	20.0(19.3–20.7)	522/4819	10.8(10.0–11.7)
50–59	1032/5524	18.7(17.7–19.7)	332/2684	12.4 (11.1–13.6)
≥60	359/1401	25.6(23.3–27.9)	107/983	10.9 (8.9–12.8)
P	0		0.01	

We further analyzed the prevalence of single and multiple HPV infections in different age groups. In the outpatient women, the peak prevalence for both single and multiple HPV infection was observed in the participants aged < 20 years. The lowest prevalence of single HPV infection in outpatients was observed in 20–29 age group, while the lowest prevalence of multiple HPV infection was observed in 30–39 age group. However, it was different from those of the healthy women where the highest prevalence of single HPV infection was observed in 50–59 age group and the lowest was in the over 60 age group. Regarding dual HPV infection in healthy women, the highest and lowest prevalence was respectively observed in the over 60 age group and the 30–39 age group (Table 5).

**Table 5:** Prevalence of single and multiple HPV infections in different age groups

Age groups	Outpatient Women				Healthy Women			
	Total	Single (%)	Dual (%)	3 or more (%)	Total	Single (%)	Dual (%)	3 or more (%)
<20	447	90 (20.1)	42 (9.4)	32 (7.2)	0	0	0	0
20-29	9886	1267 (12.8)	330 (3.3)	136 (1.4)	3098	248 (8.0)	67 (2.2)	12 (0.4)
30-39	13671	1859 (13.6)	368 (2.7)	98 (0.7)	8293	676 (8.2)	112 (1.4)	34 (0.4)
40-49	12339	1991 (16.1)	362 (2.9)	115 (0.9)	4819	429 (8.9)	74 (1.5)	19 (0.4)
50-59	5524	788 (14.3)	173 (3.1)	71 (1.3)	2684	266 (9.9)	55 (2.0)	11 (0.4)
≥60	1401	258 (18.4)	61 (4.4)	40 (2.9)	983	77 (7.8)	27 (2.7)	3 (0.3)
*P*		0	0	0		0.031	0.001	0.992

## Discussion

Epidemiological studies had shown that persistent cervical HR-HPV infection is correlated with an increased risk of developing a high-grade cervical intraepithelial lesion (e.g., cervical cancer) [11]. Fortunately, the successful development of HPV vaccines would help to prevent the occurrence of cervical cancer. Population-based epidemiological data would assist to develop HPV vaccines for a specific population.

Most of the previous researches on HPV epidemiology were only based on outpatient samples. Since the outpatients usually come to the hospital for some medical problems, the HPV prevalence is different from that of healthy women who participate in normal medical examinations. In our study, both outpatients and healthy participants were recruited from Jiangsu, China. In the 45,736 outpatient women, the prevalence of HPV was 17.7% and the HR-HPV prevalence was 15.7%. Compared with similar studies in China, the prevalence in our study was higher than that in Beijing (9.9%) [12] and Yunnan (12.9%) [13], but lower than that in Fujian (38.3%) [14] and Zhejiang (20.54%) [15]. The different HPV prevalence might be explained by different economic conditions, cultural diversity, geographical regions and survey period. Regarding the 19,877 healthy women, the overall HPV prevalence and HR-HPV prevalence was 10.6 and 10.1%, respectively. It was fully explained that more attention should be paid to the healthy women infected with HR-HPV.

The analysis of HPV prevalence showed that the most commonly identified HR-HPV genotypes were HPV 16, 58, 52, 39 and 18 among the outpatient women. It was different from the healthy women, where HPV 58, 16, 52, 39 and 56 were the five most commonly detected HR-HPV genotypes. The present study showed that HPV16, 58 and 52 were the top three detected HR-HPV genotypes in the two populations, which was similar to the other studies conducted in China, despite the prevalence varied widely [13, 16, 17]. However, it was different from the result of a meta-analysis which summarized global reports and indicated that HPV16, 18 and 45 were most commonly detected genotypes [18]. With a relatively lower prevalence for HPV 18, the vaccines against HPV16, 58 and 52 should be developed for Chinese women.

In the outpatient women with multiple HPV infection, the most commonly identified combinations of HPV types were HPV16 + 58, HPV52 + 58 and HPV39 + 52. While the top three combinations of types were HPV16 + 52, HPV52 + 58 and HPV39 + 58 for the healthy women. It was easy to note that these top three multiple-type infections were the different combinations of HPV 16, 39, 52 and 58. We also found that the vaccines targeting HPV 16/39/52/58 will protect more women in East China, when four HPV types were included in vaccines (Additional file 1: Table S1). If three HPV types were included, HPV 16, 52 and 58 were the recommended combination. When two HPV types were included, HPV 16 and 58 were the recommended combination. 4.6, 3.9 and 2.9% women will be protected by vaccines targeting HPV 16/39/52/58, HPV 16/52/58, and HPV 16/58, respectively. Because the 2v, 4v, and 9v HPV vaccines on the market do not provide protection against type 39, and the different prevalence of HPV types was observed between women in China and the other countries, it is necessary to develop HPV vaccines specific for Chinese women. The HPV vaccines that covered the four additional HR-HPV types (HPV16/39/52/58) may be more meaningful for a HPV vaccination program in China.

The peak prevalence rate of overall HPV, single HPV and HR-HPV was observed in healthy women aged 50–59 years. This was very different from the findings in outpatients of China. Previous studies have reported that HPV prevalence had two peaks in terms of age, while the peak age varied in different studies [19–21]. The two peaks were generally found in younger and menopausal women. Wang et al. found that HR-HPV infection showed higher rates in younger groups and lower rates in middle age groups and the highest prevalence in individuals aged 15–19 years [22]. Tang et al. reported that individuals aged 21–40 years had highest HPV prevalence [23]. Our data suggested that the highest HPV prevalence was found in outpatient women at the ages of < 20 or ≥ 60 years.

## Conclusion

In the present study, we presented the data on the prevalence characteristics of HPV in both outpatient women and healthy women who were recruited from Jiangsu province, east of China. Such data could guide interventions to control prevalence of HPV. Vaccines against HPV58/16/52/39 should be developed for Chinese women specifically. 50–59 years old women had highest HPV prevalence and should be paid more attention.

## Additional files

Additional file 1: **Table S1.** The estimated percentage of women who will be protected by the vaccines targeting different HR-HPV types (XLSX 56 kb)

Additional file 2: **Table S2.** The all multiple HPV infection mode in participants. (XLSX 29 kb)

## Abbreviations

CI: Confidence interval
HPV: Human papillomavirus
HR: High risk
LR: Low risk
UR: Undetermined risk
